# A case of massive hepatic infarction in severe preeclampsia as part of the HELLP syndrome

**DOI:** 10.11604/pamj.2020.36.78.23302

**Published:** 2020-06-09

**Authors:** Linda El Allani, Said Benlamkaddem, Mohamed Adnane Berdai, Mustapha Harandou

**Affiliations:** 1 Maternal and Paediatric Critical Care Unit, Hassan II Academic Hospital, Fez, Morocco

**Keywords:** Preeclampsia, HELLP syndrome, hepatic infarction, post-partum

## Abstract

Hepatic infarction is a rare and fatal complication associated with hemolysis, elevated liver enzymes and low platelets syndrome. It can develop into fulminant liver failure and lead to death in 16% of cases. A 25-year-old woman, with unremarkable prenatal history, was sent to gynecological emergency unit for management of severe preeclampsia at 30 weeks and 4 days of pregnancy. Initial laboratory studies revealed aspartate aminotransferase at 290 U/L, alanine aminotransferase at 193 U/L and a normal value of hemoglobin, platelets and the prothrombin time. Behind the persistence of high blood pressure despite dual therapy, an emergent cesarean section was performed. However, two days after surgery, the patient accused an epigastric pain and was subsequently noted to have developed HELLP syndrome: thrombocytopenia (77000 /ul), anemia (hemoglobin 9.1 g/dL) and worsened liver injury (aspartate aminotransferase 2809 U/L; alanine aminotransferase 2502 U/L). A thoraco-abdominopelvic computed tomography (CT) was performed, which revealed massive hepatic infarction more marked on the right lobe, by showing the existence of diffuse hypodense plaques, poorly limited, not enhanced after injection, interesting all hepatic segments. The vascular permeability of the portal and subhepatic was preserved. During the surveillance, the laboratory tests worsened (hemoglobin = 4,6 g/dl; platelets count = 20000 /ul; WBC = 26000 /ul; CRP = 340 mg/l; albumin = 16 g/l, prothrombin time (PT) = 50%). The patient received antibiotics, she was transfused by red blood cells and platelets concentrates, she also received albumin with the pleural effusion drainage. The damaged hepatic areas stayed stable in control CT and the patient gradually improved here biological test, to become normal at 11 days after delivery. Hepatic infarction is an extraordinarily rare complication of preeclampsia. The diagnosis should be suspected by noting elevated liver enzymes, thrombocytopenia and typical images of hepatic infarction on abdominal CT. Early recognition and multidisciplinary management is necessary to prevent hepatic failure and death.

## Introduction

Preeclampsia is a pregnancy specific pathology, defined as new established hypertension and proteinuria occurring after the 20^th^ weeks of gestation [1, 2]. Severe forms of preeclampsia can lead to numerous complications, especially HELLP syndrome (hemolysis, elevated liver enzymes and low platelets), this entity gathers hemolysis, hepatic lesions and low platelet count [3, 4]. Extensive hepatic infarction is rarely described as a complication of HELLP syndrome, a limited number of cases have been published in literature and the true incidence still unknown. Fulminant liver failure can be a serious consequence of hepatic infarction and can compromise both maternal and neonatal vital prognosis [5]. We report the case of a patient with massive hepatic infarction associated with preeclampsia and HELLP syndrome.

## Patient and observation

It´s about a 25-year-old woman, with no remarkable pathological history (in particular no history of hypertension, autoimmune disease, thromboembolism or chronic liver disease), gravida two para one, who was referred to the gynecological emergency unit of our university hospital for hypertension neurosensory symptoms (headache and blurred vision) associated to high blood pressure and lower extremity edema, on a pregnancy estimated at 30 weeks and 4 days of gestation. First examination showed a conscious patient (Glasgow coma scale (GSC) at 15/15), osteo-tendinous and plantar reflexes were normal, otherwise no motor or sensory deficits were detected. The blood pressure was 180/110 mmHg. Furthermore, the obstetric ultrasound showed a positive foetal cardiac activity. Laboratory test results showed 4+ proteinuria, hemoglobin at 13 g/dl; aspartate aminotransferase (AST) at 290 IU/l; alanine aminotransferase (ALT) at 193 IU/l, platelets count at 383000 /uL, total bilirubin at 37 mg/L and direct bilirubin at 21 mg/L. Elseways, the renal function and the prothrombin time (PT) were normal. We administered antihypertension drugs (a bolus dose of intravenous (IV) nicardipine and PO methyldopa: 500 mg every 8 hours), and the patient was started on magnesium sulfate for seizure prophylaxis. Considering the risk of a preterm delivery, she also received betamethasone for fetal lung maturity.

**Figure 1:**
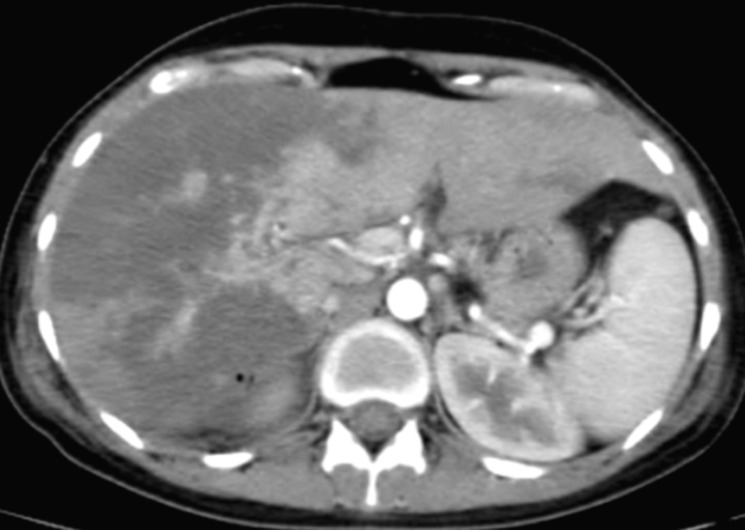
contrast-enhanced CT of the abdomen showing the presence of diffuse hypodense plaques more marked on the right hepatic lobe consistent with massive hepatic infraction

However, those symptomatic treatments were inefficient, so the decision was taken for immediate cesarean delivery indicated for maternal rescue. Within 2 days of delivery, the patient installed an intense epigastric pain, the hemoglobin dropped to 9,1 g/dl, AST rose to 2809 IU/l and ALT to 2502 IU/l, platelets count decreased to 77000 /ul, lactate dehydrogenase (LDH) was at 1209 U/L, prothrombin time (PT) was at 61% and blood glucose level was normal. The renal function was also disturbed (serum creatinine increased to 16 mg/l and urea achieved 1,58 g/l). Then, based on all above, we diagnosed the patient with HELLP syndrome and she was immediately transferred to the medical intensive care unit (MICU) for suspicion of liver subcapsular hematoma complicating the HELLP syndrome. Arriving to the MICU, the patient still had the epigastric pain. The physical examination objectified a blood pressure at 130/90 mmHg, heart beats at 140 per minute, temperature at 37.1^°^C, with sensitivity of the right hypochondrium and decreased breath sounds. The ultrasonic images showed a hyperechoic areas interesting different hepatic segments with massive hydrothorax. We completed paraclinical investigations by a thoraco-abdominopelvic CT (Figure 1), it objectified the absence of spontaneous hemorrhagic hyperdensity and the existence of diffuse hypodense plaques, poorly limited, not enhanced after injection, interesting all hepatic segments, more marked on the right hepatic lobe.

**Figure 2:**
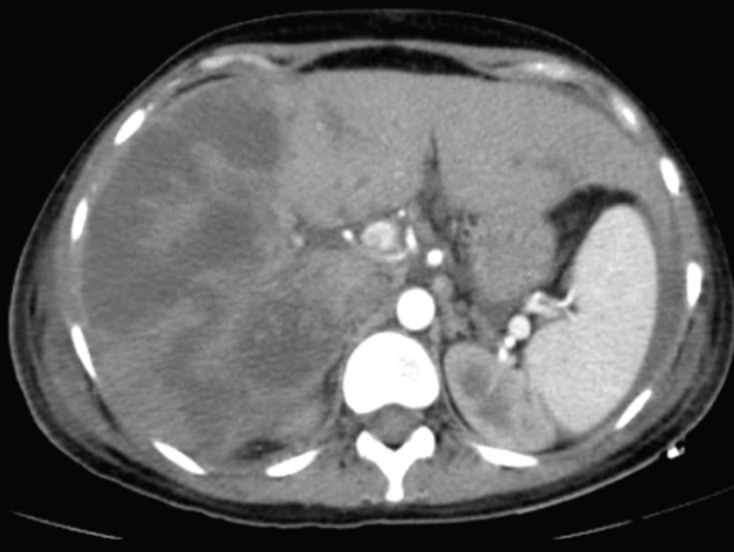
contrast-enhanced CT of the abdomen showed the damaged areas were persisted on the 15^th^ days after admission

The vascular permeability of the portal and subhepatic was preserved. Those CT images correspond to massive hepatic infarction. Otherwise, the CT scan also showed the presence of an intra-peritoneal and pleural effusions. Once the diagnosis of HELLP syndrome with massive hepatic infarction was confirmed, we continued to treat the patient with antihypertension drugs (PO nicardipine and PO methyl dopa), preventive anticoagulant treatment and proton pump inhibitor. During the surveillance, the patient installed a polypnea with an aggravation of pleural effusion on chest X-ray, she became febrile and the biological features worsened (hemoglobin = 4,6 g/dl; platelets count = 20000 /ul; WBC = 26000 /ul; CRP = 340 mg/l; albumin = 16 g/l, prothrombin time (PT) = 50%). Then, the patient received antibiotics (4g piperacillin / 0.5g tazobactam given every six hours for 14 days and 15 mg/kg amikacin per day as a single daily dose for 3 days), she was transfused two units of packed red blood cells and seven units of platelets concentrates, she also received albumin 20 g/day with the pleural effusion drainage. Within 6 days, the patient has improved clinically, liver function tests began to downtrend by postoperative day 5, the biological results were normalized within 11 days, the damaged hepatic areas stayed stable for 15 days as shown by a control CT scan (Figure 2). So, at day 17 the patient was discharged with maintained follow-ups.

## Discussion

The HELLP syndrome was described for the very first time by Weinstein in 1982, who designed a particular group of preeclamptic patients with hemolysis, elevated liver enzymes and low platelet count and considered it as a special form of severe preeclampsia [4]. The incidence of HELLP syndrome is between 10 and 20% of patients with severe preeclampsia, in 30% of times it occurs in the post-partum period [3, 6] and so was the case for our patient. The risk factors for this pathology are: multiparity, maternal age above 25 years and the white race. Moreover, HELLP syndrome is also reported to occur more frequently in patient with antiphospholipid antibody syndrome (APLS). The pathogenesis of the HELLP syndrome still unknown and the supposed mechanisms are difficult to differentiate from those of preeclampsia. Thus, the HELLP syndrome results from a disseminated microangiopathy, which is the consequence of a trophoblastic implantation defect. This defect leads to a local imbalance of expression between vasoconstrictor and vasodilator mediators, leading to placental ischemia. Placental ischemia is thought to be the cause of disseminated microangiopathy through two phenomena: placental production and release of free radicals, sFlt1 and syncytial microvilli into general circulation [7, 8]; activation of neutrophils and their fixation on the vascular endothelium [9, 10].

The cumulative effect of these two phenomena leads to systemic vasoconstriction. This worsens placental ischemia and is responsible for improper activation of the coagulation process. The diagnosis of HELLP syndrome is purely biological, associating hemolytic anemia, thrombocytopenia (< 100000 /ul) and elevation of aminotransferases. However, some clinical manifestations, especially right upper quadrant pain, are described in women with HELLP syndrome, while others may remain asymptomatic. A study of the literature reveals only a limited number of HELLP syndrome complicated by hepatic infarction. This rare complication is very severe and leads to death in 16% of cases [11]. Hepatic infarction lesions occurring in the context of a HELLP syndrome are integrated into a systemic process following the development of disseminated intravascular coagulation. The histological translation of this is represented by hepatocytic necrosis and hemorrhage foci, associated to fibrinoid deposits in the sinusoids. The clinical symptoms of hepatic infarction include right upper quadrant pain and fever [12] as with our patient.

Other authors report also the existence of jaundice [12]. The pain of the right hypochondrium can be explained by the hepatic parenchymal infarction, or more frequently by the presence of an intra parenchymatous or subcapsular hematoma, whose complication is the intraperitoneal rupture what makes all the gravity of the disease [13]. Biologically, this pathology is reflected by suddenly increased liver aminotransferases [12], often between 30 and 60 times the normal value, it goes with our findings. Both clinical manifestations and laboratory features are nonspecific and may be found in other diseases such as viral hepatitis, drug induced hepatitis, acute fatty liver as well as Budd-Chiari syndrome. Computed tomography scan is indeed the most sensitive method for the diagnosis of liver infarction, for the search of hemorrhage complications (intraparenchymal hemorrhage, subcapsular hematoma and hemoperitoneum) as well as the establishment of differential diagnosis. The hepatic ischemic damage takes the form of hypodense, well-defined, wedge-shaped lesions with no mass effect on adjacent structures.

These lesions, of non-systematized topography, predominate at the periphery of the right liver and do not enhance after injection. This is in perfect harmony with our imaging results. As soon as the diagnosis of HELLP syndrome is suspected, the management must be conducted in a structure comprising both maternal and neonatal intensive care unit. Etiological treatment of HELLP syndrome is based on the interruption of pregnancy [14], in our case this syndrome only appeared after the extraction indicated initially for severe preeclampsia. In addition to antihypertensive drugs, the management consists also in the transfusion of blood products. Platelet transfusion is only indicated if sever thrombocytopenia (<50000 /ul) with active bleeding or hemorrhage risk. The transfusion of red cells is indicated for severe or poorly tolerated anemia. In the case of coagulopathy often seen with HELLP syndrome, the coagulation factors must be corrected with fibrinogen and fresh frozen plasma [14]. Many studies advocate for the use of corticosteroids in order to improve clinical signs and biological features of HELLP syndrome [15, 16]. However, the literature on this subject is discordant. In the case of our patient they were not administered. Some publications reports the hepatic transplant as an ultimate treatment for HELLP syndrome most destructive hepatic complications [17, 18].

## Conclusion

Extensive hepatic infarction is a catastrophic finding of pregnancy in patients with HELLP syndrome. It is difficult to do diagnosis since findings and symptoms are non-specific. Clinicians should always pay attention to pregnant women referring with the complaints of hypertension, epigastrium or shoulder pain, including patients at postpartum early period.
